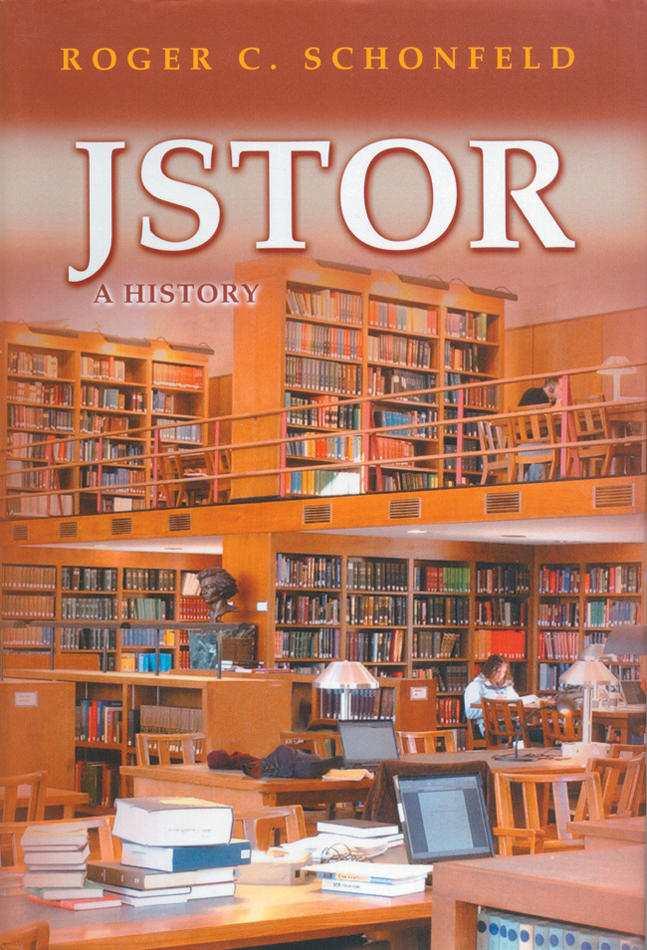# Looking from the Past to the Future

**DOI:** 10.1371/journal.pbio.0020010

**Published:** 2004-01-20

**Authors:** Frederick J Friend

## Abstract

The history of one of the world's first electronic archives for scientific journals

JSTOR is successful for reasons its founders did not intend. Bill Bowen's inspired vision was of a solution to libraries' ever-voracious demands for space to house paper volumes. The idea was that libraries could save space by removing volumes available in electronic format. Few libraries have discarded the volumes digitised in JSTOR, but many libraries without the paper volumes have been able to offer their users access to the important journal runs JSTOR has digitised. Paper holdings have not decreased dramatically, but electronic holdings have increased. So a space-saving service became an access service.

As an access service, JSTOR is a creation of its time. Understandable though the decision to use page images may have been eight years ago, future user-friendly access requires searching capabilities across full-text, which page images cannot supply. Likewise, the decision to digitise the back-runs of around 100—now 218—paper journals was a bold decision at the time, but the future for access to journal literature lies in electronic versions of thousands rather than hundreds of titles, both current and retrospective. When we reach that point, JSTOR will still have a valued place in the content on offer, but it is difficult to see JSTOR providing thousands rather than a few hundred titles. Its technical solutions and financial models look dated as both subscription-based and open-access publishers improve their services to authors and to readers. As the number of journal articles accessible over the networks increases, JSTOR will be seen as a small-scale pioneer from which we learned valuable lessons.

Roger Schonfeld ends his very detailed description of JSTOR with a chapter on ‘Lessons Learned’, many of which are relevant to current access initiatives. The need for grant funding to launch any such initiative has to be accompanied by a sound business plan to ensure long-term economic viability. JSTOR has achieved that transition, and its success provides a model for others. Much of the credit must go to JSTOR's enterprising president, Kevin Guthrie, who found the quickest way through the maze of conflicting advice—much of which could have resulted in JSTOR's reaching a deadend—and convinced the library and publishing communities to buy into a product that was only a promise. Meeting user needs for easy access to high-quality content was the key to the fulfilment of that promise. JSTOR's public image is of quality in depth—long runs of core journals—and that image has to become the hallmark of the new open-access initiatives as they develop.

It is understandable that some mistakes were made on the way. The difficulty that JSTOR financial planning had in coming to terms with consortial purchases delayed its growth as an access service. Although selling to consortia of academic libraries may not have improved JSTOR's financial position in the short-term, consortia are a route to spreading access and therefore securing longer-term financial stability (as the major publishers have realised through their ‘Big Deals’ in selling hundreds of journals to hundreds of libraries in a consortium). Some opportunities were also delayed—not lost—through too slow an adaptation of the JSTOR purchasing model for selling outside the United States, the United Kingdom being the exception. The UK deal was with JISC, the Joint Information Systems Committee of the UK Higher Education Funding Councils, acting more as a negotiating agent than a consortium, and this model could have been applied in other countries. More countries would have valued access to JSTOR earlier, but the approach to non-US deals had to be imaginative. For all vendors, there has to be an understanding of the political, social, economic, and educational structure of the country into which the product is being sold, an understanding that takes time to acquire but that pays dividends. Open-access publishers do not have to sell their product to users of their journals, but local knowledge is essential in ‘selling’ their services to authors. The globalisation of publishing has combined with the globalisation of the networks and with the globalisation of research to provide opportunities for high-quality research conducted outside North America and Western Europe to be published in peer-reviewed open-access journals more readily than in the traditional subscription-based journals.

Roger Schonfeld's book draws out many of the significant points about JSTOR's place in the history of electronic publication through a minute examination of the process leading to JSTOR as it is today. There is so much detail in the book that the reader may feel that its comprehensiveness cannot be questioned, but one small omission of which I have personal knowledge makes me question the value of so much detail. The omission concerns the interest by my institution, University College London, in joining JSTOR before the JISC deal was considered. Not a detail of world-shattering significance, but it does illustrate the fact that outside the United States, as well as within, the early interest in JSTOR came from individual institutions rather than from consortia. I sympathise with Roger Schonfeld in attempting to write such a comprehensive history, but what is the point of appearing to be comprehensive when comprehensiveness is an impossible goal? Would a briefer history have been just as valuable?

Leaving aside quibbles and caveats about the book and about JSTOR, this remains a fascinating and instructive history of an important and ground-breaking initiative. Bill Bowen's vision may not have developed in quite the way he expected, but the ‘bottom-line’ is that the vision did become a successful reality. The problem of ever-expanding libraries has not gone away in the ten years since JSTOR was conceived, but the ultimate solution—the availability of electronic content—has become closer, and JSTOR's success has encouraged others to develop services that are more in accord with 2003 than 1993. One lesson Roger Schonfeld does not draw out is the pace of change in electronic publishing, and if so much has been achieved since 1993, what promise is held out by the next ten years'!

## 

**Figure:**